# Authors’ response: Added value of backward contact tracing for COVID-19

**DOI:** 10.2807/1560-7917.ES.2024.29.4.2400047

**Published:** 2024-01-25

**Authors:** Timo Louis Boelsums, Maaike Buskermolen, on behalf of all co-authors of the original article

**Affiliations:** 1 Department of Infectious Disease Control, Public Health Service Rotterdam-Rijnmond, Rotterdam, the Netherlands; 2 https://www.eurosurveillance.org/content/10.2807/1560-7917.ES.2023.28.41.2200916

**Keywords:** COVID-19, SARS-CoV-2, backward contact tracing, pandemic preparedness, public health response, infectious disease control

**To the editor:** We thank Fraser et al. for their thoughtful suggestions about our study, in which we conclude that the addition of manual backward contact tracing (BCT) specifically to control the COVID-19 pandemic did not provide added value in our study setting [1]. Fraser et al. elaborated on some of the explanations we have described in our discussion and added some additional and interesting explanations for our findings.

We agree with Fraser et al. that the lockdown during the period of our study (February–March 2021) may have affected the effectiveness of BCT in our study in an important way. In our discussion, we mentioned another study on BCT by Raymenants et al. [2]. The study period was extended beyond the lifting of stringent lockdown measures. In this extended period, the effectiveness of BCT seemed to improve substantially.

In the letter to the editor, it was correctly stated that BCT should have the most value in uncovering transmission chains not yet known to public health authorities. Overdispersion is an important factor in the effectiveness of BCT. We agree that the degree of overdispersion, which can vary by severe acute respiratory syndrome coronavirus 2 (SARS-CoV-2) variant, affects the expected added value of BCT, as suggested by Endo et al. [3]. In addition, the term 'backward contact tracing' is used in various implementation methods, and we chose to follow the method suggested in the theoretical models mentioned in our article.

As proposed in these models, BCT can be implemented by identifying an unknown source case and then identifying the additional contacts found through this source. We agree with Fraser et al. that testing asymptomatic cases could have resulted in tracing more positive cases. However, we described in our discussion that it was difficult in practice to identify contacts of asymptomatic source cases, as it is difficult to determine the infectious period of the source case. We also excluded source locations from our study because of practical difficulties. In anonymous settings such as supermarkets and public transport, identifying source cases and their contacts would have been difficult.

For settings different from those in our study, we strongly agree with Fraser et al. that BCT may be more successful. For instance, when respiratory pathogens characterised by over-dispersed transmission (particularly for initial pandemic variants), during periods of low incidence (before and after pandemic waves) and outside of lockdown periods. This could also help in gathering additional information in the early stages of a pandemic. We thank the authors for their suggestion that efforts to perform BCT have the potential to inform national control policies.
